# Comparative testicular transcriptome of wild type and globozoospermic *Dpy19l2* knock out mice

**DOI:** 10.1186/2051-4190-23-7

**Published:** 2013-09-03

**Authors:** Thomas Karaouzène, Michèle El Atifi, Jean-Paul Issartel, Marianne Grepillat, Charles Coutton, Delphine Martinez, Christophe Arnoult, Pierre F Ray

**Affiliations:** Université Joseph Fourier, Grenoble, F-38000 France; Laboratoire AGIM, CNRS FRE3405, Equipe “Génétique, Infertilité et Thérapeutiques”, La Tronche, F-38700 France; Team7 Nanomedicine and Brain, INSERM U836, Grenoble, France; Institut des Neurosciences, Université Joseph Fourier Grenoble, Kragujevac, France; Clinical Transcriptomics and Proteomics Platform, Centre Hospitalier Universitaire et Grenoble Institut des Neurosciences, Grenoble, CNRS, Grenoble, France; CHU de Grenoble, UF de Biochimie et Génétique Moléculaire, Grenoble cedex 9, F-38043 France; CHU de Grenoble, Département de Génétique et Procréation, Grenoble cedex 9, F-38043 France

**Keywords:** Male infertility, Globozoospermia, Spermatogenesis, Dpy19l2, Transcriptome

## Abstract

**Background:**

Globozoospermia is a male infertility phenotype characterized by the presence in the ejaculate of near 100% acrosomeless round-headed spermatozoa with normal chromosomal content. Following intracytoplasmic sperm injection (ICSI) these spermatozoa give a poor fertilization rate and embryonic development. We showed previously that most patients have a 200 kb homozygous deletion, which includes *DPY19L2* whole coding sequence. Furthermore we showed that the DPY19L2 protein is located in the inner nuclear membrane of spermatids during spermiogenesis and that it is necessary to anchor the acrosome to the nucleus thus performing a function similar to that realized by Sun proteins within the *LINC*-*complex* (Linker of Nucleoskeleton and Cytoskeleton). SUN1 was described to be necessary for gametogenesis and was shown to interact with the telomeres. It is therefore possible that Dpy19l2 could also interact, directly or indirectly, with the DNA and modulate gene expression during spermatogenesis.

In this study, we compared the transcriptome of testes from *Dpy19l2* knock out and wild type mice in order to identify a potential deregulation of transcripts that could explain the poor fertilization potential of *Dpy19l2* mutated spermatozoa.

**Methods:**

RNA was extracted from testes from *DPY19L2* knock out and wild type mice. The transcriptome was carried out using GeneChip® Mouse Exon 1.0 ST Arrays. The biological processes and molecular functions of the differentially regulated genes were analyzed with the PANTHER software.

**Results:**

A total of 76 genes were deregulated, 70 were up-regulated and 6 (including *Dpy19l2*) were down-regulated. These genes were found to be involved in DNA/RNA binding, structural organization, transport and catalytic activity.

**Conclusions:**

We describe that an important number of genes are differentially expressed in *Dpy19l2* mice. This work could help improving our understanding of *Dpy19l2* functions and lead to a better comprehension of the molecular mechanism involved in spermatogenesis.

**Electronic supplementary material:**

The online version of this article (doi:10.1186/2051-4190-23-7) contains supplementary material, which is available to authorized users.

## Résumé

**Contexte:**

La globozoospermie est caractérisée par la présence dans l’éjaculat de près de 100% de spermatozoïdes ronds et dépourvus d’acrosome qui présentent un contenu chromosomique normal. L’injection intracytoplasmique (ICSI) de ces spermatozoïdes donne cependant un taux de fécondation et de développement embryonnaire particulièrement bas. Nous avons montré précédemment que la plupart des patients globozoospermes présentent une délétion homozygote de 200 Kb qui inclue la totalité de la séquence codante du gène *DPY19L2*. De plus nous avons montré que la protéine *DPY19L2* était localisée dans la membrane interne des noyaux des spermatides pendant la spermatogénèse et qu’elle est nécessaire pour fixer l’acrosome au noyau, réalisant ainsi une fonction similaire à celle des protéines Sun au sein du complexe LINC (Linker of Nucleoskeleton and Cytoskeleton). Il a par ailleurs été montré que SUN1 était nécessaire à la spermatogénèse et que cette protéine interagit avec les télomères chromosomiques. Il est donc possible que *Dpy19l2* interagisse également, directement ou indirectement avec l’ADN et module l’expression génique lors de la spermatogénèse. Dans cette étude nous avons donc comparé le transcriptome de testicules de souris invalidées (KO) pour le gène *Dpy19l2* à celui de souris sauvage afin d’identifier une éventuelle dérégulation génique qui pourrait expliquer le faible potentiel reproductif des spermatozoïdes globozoocéphales.

**Méthode:**

L’ARN a été extrait de testicules de souris KO pour *Dpy19l2* et de souris sauvages. Le transcriptome a été réalisé en utilisant des puces d’expression ® Mouse Exon 1.0 ST Arrays. Les processus biologiques et les fonctions des gènes dérégulés ont été analysés en utilisant le logiciel PANTHER.

**Résultats:**

Un total de 76 gènes a été identifié comme étant dérégulés, 70 gènes étaient surexprimés et 6 (incluant Dpy19l2) étaient sous-exprimés. Il s’agit de gènes principalement impliqués dans des interactions avec des acides nucléiques (ADN/ARN), ou ayant un rôle structural, dans le transport, ou présentant une activité catalytique.

**Conclusions:**

Cette étude nous a permis d’identifier et de décrire un nombre important de gènes exprimés de manière différentielle chez les souris KO pour *Dpy19l2*. Ce travail peut permettre d’améliorer notre compréhension des fonctions de Dpy19l2 et peut contribuer à obtenir une meilleure compréhension des mécanismes moléculaires nécessaire à la spermatogénèse.

## Background

A recent study supported by the World Health Organization indicates than in 2010, an estimated 48.5 million couples worldwide were unable to have a child after five years [1]. Male factors are believed to be responsible for 30-50% of all infertility cases, but micro deletions of the Y chromosome are the only genetic defects altering human spermatogenesis, which are diagnosed routinely.

To be able to fertilize the oocyte, the spermatozoon needs to cross the zona pellucida (ZP), which is a glycoprotein layer surrounding the oocyte. The acrosomal reaction (AR), during which the acrosome (a giant vesicle of secretion) releases its content, plays an important role in the fertilization process. Enzymes released from the acrosome locally digest and soften the ZP so that the spermatozoon can penetrate deeper and fertilize the oocyte. The acrosome, a highly specialized organelle found only in sperm, is tightly bound to the nucleus via the acroplaxome (a network of proteins including keratin 5 and β-actin) [2].

Globozoospermia is a severe teratozoospermia characterized by the presence of 100% of round-headed spermatozoa devoid of acrosome. Men with globozoospermia have a primary infertility due to this absence of acrosome, which prevents their sperm from fertilizing the oocytes in vivo [3]. Spermatozoa from globozoospermic patients have near normal levels of aneuploidy but give a poor fertilization rate and embryonic development even when performing Intra Cytoplasmic Sperm Injection (ICSI) [3]. Studies by immunocytochemistry showed that most round headed sperm lacked the phospholipase zeta protein (PLCzeta), a protein normally located around a the sperm’s head [4–7] and required to induce oocyte intracellular calcium oscillation and oocyte activation [8, 9]. It has therefore been postulated that it is the absence of PLCzeta which might be responsible for the poor fertilization potential of round-headed spermatozoa [10]. In the course of this work we wanted to assess if the absence of PLCzeta in round-headed spermatozoa results from a transcriptional repression of the gene and if other transcriptional deregulations could also contribute to the poor fertilization potential of these gametes.

The syndrome of globozoospermia was first described in the seventies [7, 11] and cases have been described regularly since [12–20]. Familial cases rapidly pointed to a genetic cause for this syndrome. In the recent years, *SPATA16* has been described to be involved in globozoospermia [21]. We demonstrated recently that DPY19L2 was in fact the main locus associated with globozoospermia as 15 out of 20 analysed patients presented a 200 Kb homozygous deletion removing the entire gene [22]. We then identified DPY19L2 point mutations and heterozygous deletions and demonstrated that 84% of the 31 globozoospermic patients analysed had a molecular alteration of DPY19L2 [23]. We finally confirmed that the recurrent deletion observed in a majority of men with globozoospermia was caused by non-allelic homologous recombination (NAHR), between two highly homologous sequences, or low-copy repeats (LCR), located on each side of DPY19L2 [24].

We previously characterized *Dpy19l2* Knockout mice (*Dpy19l2*^-/-)^ and showed that these mice present the same phenotype than men carrying mutations in *DPY19L2*, ie round-head spermatozoa without acrosome. It also permitted us to determine that i.) *DPY19L2* is located in the inner nuclear membrane of wild type mouse spermatids, ii.) *DPY19L2* is required for acrosome attachment to the nucleus and iii.) the detachment of the acrosome in *Dpy19l2*^-/-^ mice prevents correct anchoring of the manchette. Moreover we described that *SUN5* and *DPY19L2* partially colocalized in transfected HEK cells [25]. SUN-domain proteins are known to interact with chromosome-binding proteins and various KASH-domain partners to form SUN-domain-dependent 'bridges’ across the inner and outer nuclear membranes. These bridges physically connect the nucleus to every major component of the cytoskeleton [26]. SUN1, one of the members of the family, was described to be necessary for gametogenesis and was shown to interact with the telomeres [27]. We can hypothesize that *Dpy19l2* could interact directly or indirectly with the DNA and thus have an effect on the regulation of transcription. It is thus possible that the absence of *Dpy19l2* could cause some modification in the germ cell transcription pattern.

The goal of this study was to determinate if *Dpy19l2* knock out mice present significant testis transcriptional modifications compared to wild type and in particular modifications that may explain the poor success rate encountered by globozoospemic patients following ICSI- IVF.

## Methods

### Ethical statement

Animal housing and sacrificing was in accordance with French guidelines on the use of animals in scientific investigations with the approval of the local Ethical Committee.

### Animals

Dpy19l2 knock out mice were obtained from Mutant Mouse Regional Resource Center, University of California, Davis, CA. The mouse colony used in this study was initiated from two couples. The first one consisted of an heterozygous female and a wild type male. The second was composed of two heterozygous mice for the Dpy19l2 deletion. Reproduction of these two couples achieved wild type, heterozygous and homozygous *Dpy19l2* deleted mice. Mice were sacrificed at 2 months old, which means that they were pubescent and that their reproductive organs were fully established. A total of four animals were sacrificed. RNA was extracted from two homozygous WT and two homozygous KO animals.

### Genotyping PCRs

Genotyping was done on DNA isolated from tail biopsies. Tail biopsies (ca. 2 mm in length) were digested in 200 μl lysis *Direct PCR LYsis Reagent* (Tail) (Viagen Biotech inc, CA, USA) and 0,2 mg of proteinase K for 12–15 hours at 55°C and 1 hour at 85°C. The DNA was directly used for PCRs.

**Figure 1:**
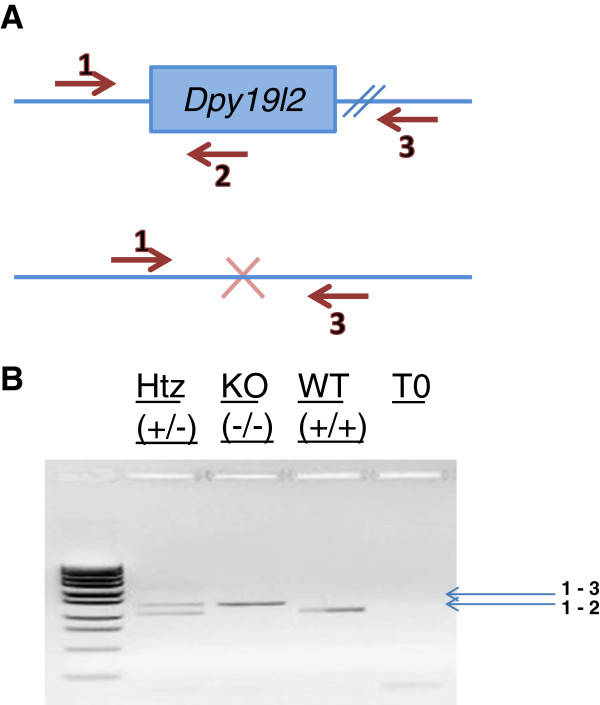
**Strategy for Dpy19l2 KO mice genotyping. A)** Scheme of the *Dpy19l2* alleles and primers (red arrows) used for their detection. Primer sequence is as follow: 1:GAAGGCTACACCTCTTGCA, 2:GCTGCAGCAACGACCACTTC; 3:CCTAGGAATGCTCGTCAAGA. **B)** Examples of PCRs with (from left to right) *Dpy19l2*+/- mouse, *Dpy19l2*-/- mouse, *Dpy19l2*+/+ mouse and water (control).

PCR was done for 35 cycles, with an annealing temperature of 57°C, and an elongation time of 60 seconds at 72°C. The primers used are described in Figure 1. PCRs products were separated on agarose gel electrophoresis. Genotypes were determined according to the migration pattern (Figure 1).

### Tissue collection

Mice were sacrificed and testes were collected. Tissues were snap frozen in liquid nitrogen prior storage at -80°C. Two mice in each group were used for the micro-array analysis.

### RNA extraction

Total RNA was extracted from tissues using mirVana isolation kit™ (Ambion, Applied Biosystems, Foster City, CA) as per the manufacturer’s instructions. RNA purity and quantity was assessed using the NanoDrop c1000 (ThermoFisher Scientific, Waltham, USA). Quality was determined by both evaluation of the integrity of rRNA bands using RNA Nano 6000 kit (Bio-Analyser, Agilent Technologies, Palo Alto, CA) and absorbtion readings ant 260 and 280 nm. For detail see Additional file 1: Table S1.

### Array hybridization

For each group, two biological replicates were used. The replicates came from four separate RNA extractions: two from homozygous WT and two from homozygous KO animals. cDNA synthesis, amplification, enzymatic fragmentation and biotinylation were performed using the Ambion WT Expression Kit (Ambion, Austin, TX, USA). Samples were hybridized to Affymetrix GeneChip® Mouse Exon 1.0 ST Arrays as per the manufacturer’s instructions. The Affimetrix Mouse Exon 1.0 ST array, contains probe sets for 35,557 genes. Briefly, 5 μg of fragmented biotinylated ssDNA was hybridised for 16 hrs at 45°C, 60 rpm to the array chip on a GeneChip® Hybridization Oven 640. After 16 hrs, GeneChips® were washed on a GeneChip® Fluidics station 450 using the washing script Prime 450 with buffers and stains supplied with the GeneChip Hybridisation Wash and Stain Kit from Affymetrix.

### Data acquisition and analysis

Data was acquired on a GeneChip® Scanner 3000 7G and .CEL file generation performed using AGCC. Expression Console with Robust Multi-chip Average (RMA) was used initially to extract probe intensity data. RMA background correction was applied including pre-background adjustment for GC content and quantile normalization across all chips in the experiment. Probe data was log2 transformed.

### Gene level expression analysis

Two separate experiments (experiment 1 and 2) were carried out, each time with one testis from homozygous wild type and homozygous KO mice. Hence for each gene a total of two values were obtained in WT (Dpy +/+ (1) and (2)) and KO mice (Dpy -/- (1) and (2)).

For each gene transcripts we calculated 4 ratios corresponding to the 4 possible combinations

**Figure 2:**
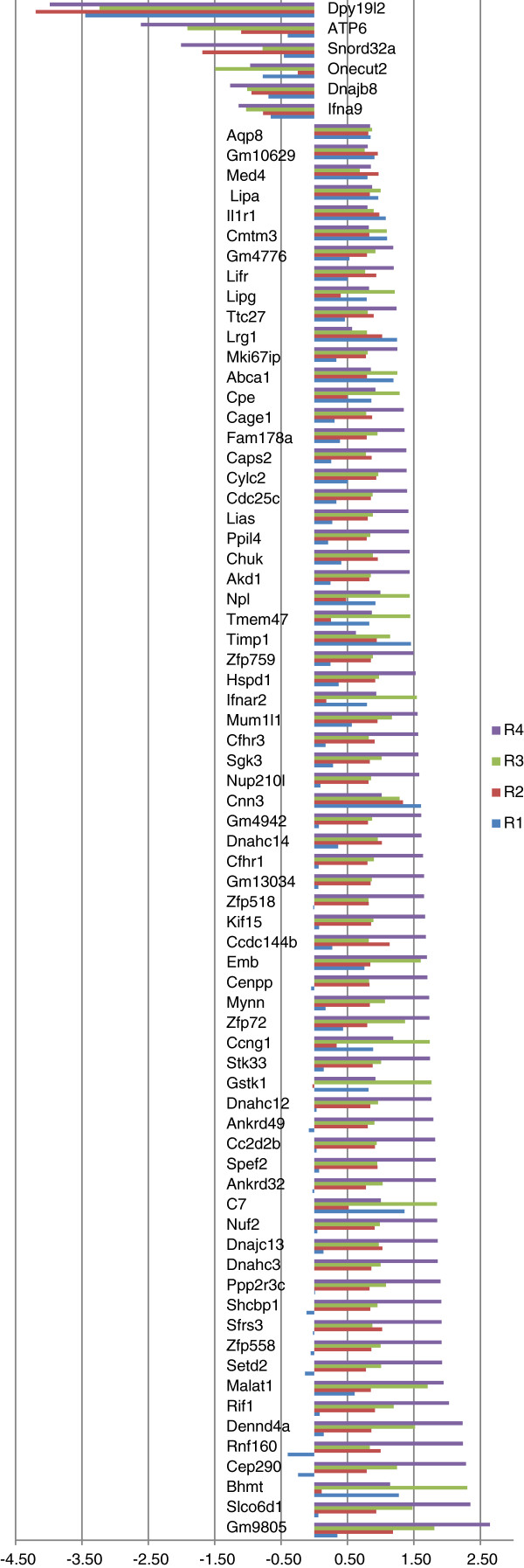
**Histogram of the log2 ratio of all deregulated transcripts.** Genes that are upregulated in Dpy19l2 KO mice have positive values while genes that are downregulated have negative values. Values can be seen in Additional file 2: Table S2.

For each gene, if at least three of these ratios appeared > = 1.7 fold up or down, the transcript was considered to be significantly differentially expressed. These values and the log2 ratio for all deregulated genes are shown in Additional file 2: Table S2. The histogram of the log2 ratio of each deregulated gene is shown in Figure 2.

### Gene ontology analysis

The lists of genes expressed differently in *Dpy19l2-/-* mice were imported into PANTHER (http://www.pantherdb.org/) to identify the biological process, molecular functions and gene networks significantly deregulated in Dpy19l2^-/-^ testis compared to WT controls.

## Results

### Gene expression profile

Array hybridization was performed with the Affimetrix Mouse Exon 1.0 ST array, which contains probe sets for 35,557 genes. Of these, we identified that 76 genes had a level of testicular expression that was different between WT and *Dpy19l2*^-/-^ mice (transcripts with an expression ratio > = 1.7 fold up- or down regulated). Among them, 6 genes were underexpressed and 70 genes were overexpressed (Figure 2 and Additional file 2: Table S2). As expected *Dpy19l2* was found part of the down-regulated genes, thus validating the experimental approach we used. Interestingly, we did not observe any difference in the expression level of PLCzeta in the testes from KO and WT mice.

### Panther gene ontology analysis

**Figure 3:**
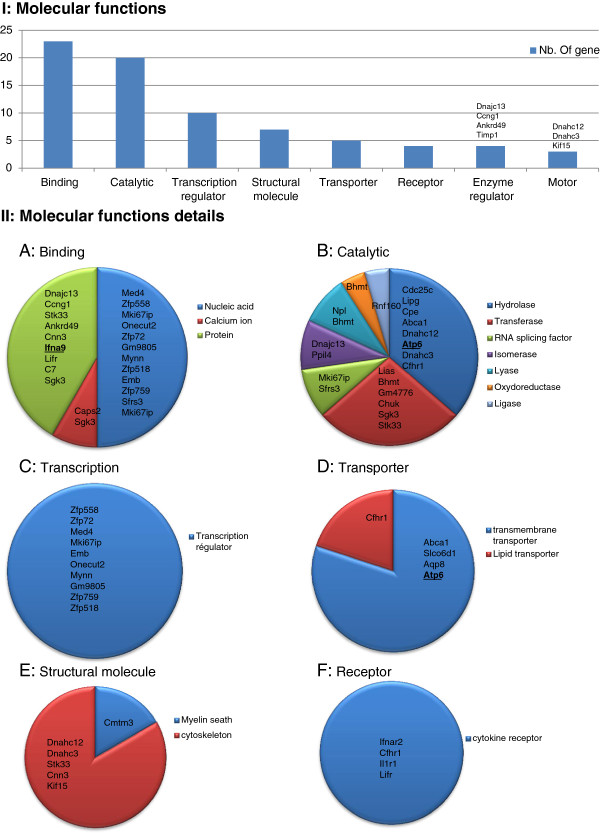
**I, Histogram presenting all PANTHER molecular function of genes that are deregulated in** ***Dpy19l2*** ^***-/-***^ **mice testes. II**, **A**-**F**, Details of some of PANTHER molecular functions. Up-regulated genes are in bold and underlined.

The 76 genes that were differentially regulated were up-loaded into the PANTHER software (Gene List Analysis). Among them 64 were recognized by the PANTHER software. The molecular functions and biological process predictions that are generated from PANTHER are based on the direction of expression of a number of downstream genes which have been previously shown to be associated with these functions. The list of each function associated to all deregulated genes is provided in Additional file 3: Table S3. Several molecular functions were found to be enriched in the testis of *Dpy19l2*^-/-^ mice (Figure 3). Genes encoding proteins witch are able to bind nucleic acids or proteins were most frequently deregulated (23 genes), especially those encoding for protein binding to the nucleic acids (12 genes), confirming that *Dpy19l2* could interact with DNA. Other functions such as catalytic activity, transcription regulator activity, structural functions were also deregulated in the KO mice testes. Because of its location in the inner nucleus membrane, *DPY19L2* could be a bridge between the nucleus and the cytoplasm. We observed that 5 genes encoding for transporters are deregulated in KO mice: among them, four are transmembrane transporters and one is a lipid transporter. Moreover globozoospermia is characterized by structural deficiency of spermatozoon head and we see that 6 deregulated genes encode for proteins with structural molecular function.

**Figure 4:**
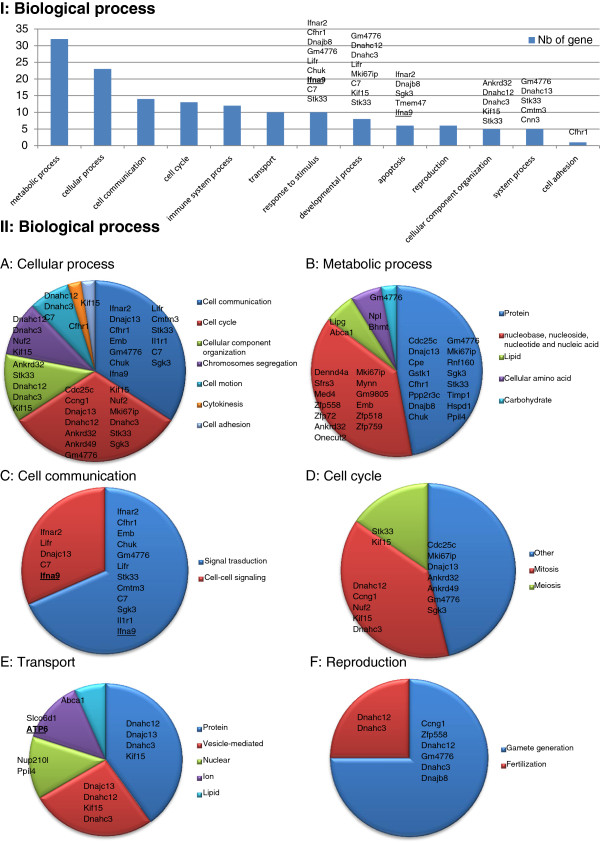
**I, Histogram presenting all PANTHER biological process of genes deregulated in** ***Dpy19l2*** ^***-/-***^ **mice testes. II**, **A**-**F**, Details of some of PANTHER biological process. Up-regulated genes are in bold and underlined.

Numerous biological processes are also deregulated in the testis of *Dpy19l2*^-/-^ mice (Figure 4). Metabolic processes and cellular processes are most often deregulated. We see that 6 genes predicted to be involved in reproduction biological process separated deregulated. Among those, no genes were described to be involved in the acrosome formation but two genes encode for dyneins and one for a protein predicted to be involved in sperm motility.

## Discussion

Spermiogenesis is the final stage of spermatogenesis. During this step, the nucleus condenses, acquires its specific shape, and the flagellum and the acrosome are formed. The acrosome is essential for the spermatozoa to cross of the ZP and is thus necessary for *in vivo* fertilization. Globozoospermia is a teratozoospermia characterised by the formation of round-head spermatozoa without acrosome. This pathology has been described to be associated with the absence of the protein PLCzeta which is also known to be essential for fertilization and oocyte activation [4–7]. We previously demonstrated that this pathology is mostly due to a homozygous deletion of the testis-specific gene *DPY19L2*[22, 23] and that DPY19L2 is expressed in spermatids and it is located only in a restricted zone of the nuclear membrane facing the acrosome.

This study revealed that 76 genes were deregulated in the testis of *Dpy19l2* KO mice. This result could be concordant with a very specific regulatory role of Dpy19l2 at the transcription level. On the other hand we note that the micro-array contains 35,557 probe-set for almost as many genes. It is therefore a small minority (0.2%) of genes that is deregulated in DPy19l2 KO mice. It is interesting to note that almost all of these genes appeared as up-regulated and that only 5 of them were down- regulated. If Dpy19l2 has a direct influence on gene regulation we can therefore say that it mainly act as a repressor of gene expression. We note that apart from Dpy19l2, which is obviously absent from the KO and is found (due to background fluorescence levels) to have a 4 fold decrease in expression compared to controls, the most down-regulated gene, *ATP6,* has a 2.6 fold decreased expression and the most up-regulated gene, Cepp, has a 2.2 fold increased expression. The observed level of transcription modifications is therefore moderate.

Dpy19l2 co-localises with SUN5 [28] and we hypothesized that SUN5 is a likely partner of Dpy19l2 [25]. In mouse, Sun1, another Sun protein, was also described to be necessary for gametogenesis and was shown to interact in the nucleus with the telomeres [27]. We observed that most of the deregulated genes (70/76) were up regulated in KO animal. We can hypothesize that Dpy19l2 could also interact, directly or indirectly, potentially via Sun5, with germ cell DNA and thus could have an effect on the regulation of transcription in spermatogenic cells. Heterochromatin is constituted by highly compact transcriptionaly repressed DNA. It regroups down-regulated genes and is particularly abundant at the periphery of the nucleus where it interacts with factors located in the nuclear lamina. We can thus speculate that Dpy19l2 could intervene during spermiogenesis to include selected genes in heterochromatin repressive domains. In the absence of Dpy19l2, these genes would not be repressed and appear as up regulated. This regulations could be a limited to selected loci as electron microscopic observations of round spermatids nuclei from *Dpy19l2* KO animals do not show any obvious difference in the abundance of heterochromatin [25].

The PANTHER software allows a classification of genes according to their predicted molecular functions (Figure 3). We see that the most represented gene function that is deregulated in Dpy19L2 mice is “binding” (23 genes). This group is divided in three sub categories: nucleic acid, protein and calcium ion (Additional file 2: Table S2). [Ca^2+^]_I_ is known to play an important role in male fertility. [Ca^2+^]_I_ signaling is the primary regulator of sperm flagellum beating and calcium intracellular rise is known to be essential for the acrosome reaction [29]. Indeed, solubilisation of the zona pelucida stimulates generation of IP_3_ in mouse sperm [30] which is known to mobilize the acrosomal Ca2+ stored to permit acrosomal reaction [31, 32]. The biochemical nature of the Ca2 + -binding sites are globally unknown but recently a calcium-binding protein has been isolated from the acrosomal membrane of bovine spermatozoa [33]. We observe that in *Dpy19l2* KO mice two calcium binding proteins are up-regulated : Caps2 and Sgk3 (Figure 2 and 3). Ten of the deregulated genes are described to encode proteins with DNA binding abilities. Although we did not find direct evidence that these encoded proteins have transcriptional regulation activities, they might be involved in the regulation of gene expression and play a role in the up- and -down regulation of some of the other genes we found to be deregulated in this transcriptome analysis.

We did not observe a down-regulation of PLCzeta that could account for its absence from round-headed sperms. This suggests that in *Dpy19l2*-/- mice *PLCzeta* is normally expressed but that the absence of *Dpy19l2* and the abnormalities it induces on sperm morphology likely prevents the correct positioning of *PLCzeta*, which is likely to be eliminated in the residual body. This hypothesis is consolidated by the fact that several studies show that treatment with a calcium ionophore improves ICSI success rates? results for men with globozoospermia [5]. We note however that fertilization and pregnancies can be achieved by ICSI on *DPY19L2* deleted men [34]. This can probably be explained by the fact that remains of misplaced PLCzeta often position near the manchette can be observed on a small proportion of round-headed sperm [6].

This study also reveals that several genes encoding for transporters were deregulated in *Dpy19l2* KO mice. Among them four are transmembrane transporters and one is a lipid transporter. We note the deregulation of the gene *Abca1*, which is expressed in mouse spermatozoa within the seminiferous tubules and the epididymis, and is a key regulator of cholesterol efflux. Depletion of the cholesterol from the cytoplasmic plasma membrane and modification of its lipid composition is one of the key events in the process of spermatozoa capacitation, which ultimately leads to the acrosome reaction and egg fertilization. Transporters and in particular those mediating cholesterol efflux, are thus particularly important. The deregulation of *Abca1* could therefore alter the physiological composition of mature sperm and contribute to the poor fertilization potential of Dpy19l2 mutant sperm.

The analyze of biological process regulated in *Dpy19l2*^-/-^ mice reveals 6 genes predicted to be involved in reproductive functions and particularly in gamete generation and fertilization. Surprisingly half of these genes code for dyneins, which are important constituents of the microtubules. The others are involved in the processes of sperm motility and cytoskeleton structure. These results can be linked to our previous observation that the absence of Dpy19l2 leads to the destabilization of both the nuclear dense lamina and the junction between the acroplaxome and the nuclear envelope. This destabilization causes a failure of the linkage of the acrosome and the manchette to the acroplaxome, a cytoskeletal plate anchored to the nuclear envelope. The manchette is a transient microtubular structure necessary during spermatid elongation. Moreover, the manchette is necessary for protein trafficking and its defects could disturb the overall distribution of proteins in spermatids [35].

## Conclusions

We showed that Dpy19l2^-/-^ induced globozoospermia altered gene expression in mice testis but the overall modifications at the transcript level remained modest. We showed that *PLCzeta* was not down-regulated in KO mice indicating that the absence of the protein observed in the sperm of globozoospermic patient is not due to a transcriptional deregulation. This likely indicates that PLCzeta cannot reach its physiological localization on round-headed spermatozoa and that it is probably lost with the cytoplasmic elimination (residual body) during spermiogenesis. We also observed that several genes encoding proteins involved in transports, and in particular Abca1, involved in the cholesterol efflux, were deregulated. This could also contribute to the poor fertilization potential of the round-headed spermatozoa. Secondary anomalies stemming from the morphological abnormalities of the sperm could also lead to a wide range of protein deregulation as exemplified by the absence of PLCzeta. A proteomic analysis of these deregulations could permit to have a functional view of the extent of the molecular anomalies present in *Dpy19l2* KO mice. Further work will permit a better comprehension of molecular mechanism involved in spermatogenesis and in the physiopathology of globozoospermia.

## Electronic supplementary material

Additional file 1: Table S1: RNA quantification. (DOC 27 KB)

Additional file 2: Table S2: Ratios of transcripts values measured in Dpy19l2 WT and KO mice. (XLS 66 KB)

Additional file 3: Table S3: PANTHER output of all deregulated genes in Dpy19l2 KO mice. (XLS 60 KB)

## Abbreviations

AR: Acrosomal reaction
DNA: Deoxyribonucleic acid
dNTP: Deoxynucleotide triphosphates
FISH: Fluorescent in situ hybridization
ICSI: Intracytoplasmic sperm injection
IVF: In vitro fertilization
Kb: Kilobase (1000 nucleotides)
KO: *Knock-out*
LCR: Low-copy repeats
LINC: Linker of nucleoskeleton and cytoskeleton
NAHR: Non-allelic homologous recombination
PCR: Polymerase chain reaction
PLC zeta: Phospholipase zeta
RNA: Ribonucleic acid
WT: Wild type
ZP: Zona pellucida.
