# Supplementary material for: Comparative testicular transcriptome of wild type and globozoospermic Dpy19l2 knock out mice
Source: Basic Clin Androl. 2013 Sep 3;23:7. doi: 10.1186/2051-4190-23-7 (PMC4346239; doi:10.1186/2051-4190-23-7)
Supplement: Supplementary file 1 — Additional file 1: Table S1: RNA quantification. (DOC 27 KB) [file 12610_2013_8_MOESM1_ESM.doc]

Additional file 1: Table S1. RNA quantification.

| Sample ID | Conc. | Unit | A260 | A280 | 260/280 | 260/230 |
| --- | --- | --- | --- | --- | --- | --- |
| Dpy19l2^+/+^(1) | 263 | ng/µl | 6,574 | 3,22 | 2,04 | 1,99 |
| Dpy19l2^+/+^(2) | 1003,3 | ng/µl | 25,084 | 12,27 | 2,04 | 2,23 |
| Dpy19l2^-/-^(1) | 771,5 | ng/µl | 19,286 | 9,212 | 2,09 | 2,2 |
| Dpy19l2^-/-^(2) | 447 | ng/µl | 11,175 | 5,508 | 2,03 | 2,21 |
